# Prevalence of Pathogens Related to Bovine Respiratory Disease Before and After Transportation in Beef Steers: Preliminary Results

**DOI:** 10.3390/ani9121093

**Published:** 2019-12-06

**Authors:** Francesco Cirone, Barbara Padalino, Daniele Tullio, Paolo Capozza, Michele Losurdo, Gianvito Lanave, Annamaria Pratelli

**Affiliations:** 1 Department of Veterinary Medicine, University of Bari, Strada per Casamassima km 3, 70010 Valenzano (Ba), Italy; francesco.cirone@uniba.it (F.C.); paolo.capozza@uniba.it (P.C.); michele.losurdo@uniba.it (M.L.); gianvito.lanave@uniba.it (G.L.); annamaria.pratelli@uniba.it (A.P.); 2 Department of Agricultural and Food Sciences, University of Bologna, Viale Fanin 46, 40127 Bologna, Italy; 3 ASL BA—Local Health Authority Veterinary Service, Via dei Mille 29, 70120 Bari, Italy; daniele.tullio@asl.bari.it

**Keywords:** journey, bovine respiratory disease, steer, welfare

## Abstract

**Simple Summary:**

Bovine respiratory disease (BRD) affects the lower respiratory tract of cattle, causing high mortality. The syndrome has a multifactorial etiology and transport seems to favor pathogen proliferation. This study investigated the prevalence of different pathogens involved in BRD, in the nasal microbiota of beef steers collected before and after a long-distance journey. A total of 56 Limousine animals were included, travelling in three different shipments, on the same route from France to southern Italy in a semitrailer, on three different days from February to April. Prior to shipment (T0) and four days after arrival (T1), two deep nasopharyngeal swabs (DNS)/steer were collected and tested by bimolecular analysis. Neither *bovine viral diarrhea virus* (BVDV) nor *bovine herpesvirus type 1* (BoHV-1) were detected. A higher prevalence of *Histophilus somni* was observed in the DNS collected during the third shipment in comparison with those registered during the first and the second one, probably due to a higher prevalence at departure. Conversely, the prevalence of *bovine coronavirus*, *bovine respiratory syncytial virus*, *Mannheimia haemolytica*, *Mycoplasma bovis* and *Pasteurella multocida* was higher on arrival in comparison with departure, confirming data reported in the literature. Overall, there were nasal microbiota changes in beef steers, with an increase in the prevalence of pathogens associated with BRD after travelling.

**Abstract:**

Bovine respiratory disease (BRD) is a serious health and economic problem in the beef industry, which is often associated with transportation and caused by different pathogens. The prevalence of *bovine herpesvirus type 1* (BoHV-1), *bovine adenovirus* (BAdV), *bovine viral diarrhea virus* (BVDV), *bovine coronavirus* (BCoV), *bovine respiratory syncytial virus* (BRSV), *bovine parainfluenza virus* (BPiV), *Pasteurella multocida*, *Mannheimia haemolytica*, *Histophilus somni*, *Mycoplasma bovis*, in the nasal microbiota of beef steers before and after the same long-distance journey from France to southern Italy was documented. Fifty-six Limousine animals of three different shipments, travelling on three different days from February to April, were included. Prior to shipment (T0) and four days after arrival (T1), two DNS/animal were collected and tested by Real Time quantitative PCR (qPCR). Univariate logistic regression was carried out, considering time and day as fixed factors and the outcome of qPCR for each pathogen as a dependent categorical dichotomous variable (positive/negative, 1/0). The fact that the number of *H. somni* positive animals were found to be higher in the third shipment than the first and second one, indicating that this pathogen was already present before loading, is relevant. The prevalence of BCoV, BRSV, *M. haemolytica*, *M. bovis*, *P. multocida* was higher at T1 than T0, suggesting that other factors, such as stress and the epidemiological status of the arrival farm, played a role. The tested animals were not treated before and after transport, and our results are in agreement with the current literature, supporting the hypothesis that the prevalence of pathogens related to BRD would increase after travelling, with an increased risk of pathogens shedding.

## 1. Introduction

Bovine respiratory disease (BRD) affects the lower respiratory tract of cattle, causing high mortality and carcasses of lower quality. The syndrome has a multifactorial etiology, including infectious agents, host and environmental factors, with particular emphasis on transport stress. The latter is indeed responsible for physiological changes that favor pathogen proliferation and invasion of tissues by opportunistic pathogens [1,2,3,4,5]. Viruses and stress-related behavior interfere with the mucociliary clearance of the respiratory tract and dysregulate the tracheal antimicrobial peptides of the innate defenses, allowing opportunistic bacteria to cause pulmonary infections [2,6]. Infectious agents of BRD include both viral and bacterial agents such as *bovine herpesvirus type 1* (BoHV-1), *bovine adenovirus* (BAdV), *bovine viral diarrhea virus* (BVDV), *bovine coronavirus* (BCoV), *bovine respiratory syncytial virus* (BRSV), *bovine parainfluenza virus* (BPiV), *Pasteurella multocida*, *Mannheimia haemolytica*, *Histophilus somni* and *Mycoplasma bovis*.

It has been shown that, to minimize the incidence of transport-related respiratory disease, antibiotics and vaccines are widely used both before and after transport [7,8,9]. However, the data on the effectiveness of these preventative methods are conflicting [9,10]. The hypothesis of this work was that there would be a change in the nasal microbiota, with an increase in the prevalence of bacteria and virus involved in BRD, in beef steers subjected to long distance transportation and not treated before the journey [1,11,12]. 

Despite the high number of trucks transporting livestock from North Europe to Italy [6], to the authors’ knowledge, there are no data available in Italy regarding the potential associations between long-distance transport and the onset of BRD after feedlot arrival. Consequently, the aim of this pilot study was to document the prevalence of the multiple pathogens involved in BRD after a long-distance travel from France to southern Italy through investigation of the nasal microbiota.

## 2. Materials and Methods

The experimental procedures were approved by the Ethics Committee of the Department of Veterinary Medicine of the University of Bari, Italy (authorization no. 16/18). A total of 56 Limousine beef steers, approximately 6–10 months of age with an initial body weight of about 400 kg, were included in this study. They travelled for about 1600 km/28 h from an assembly point in France (Malonze, 23300 La Souterraine) to southern Italy (Gioia del Colle, Bari) in a gooseneck semitrailer with two mobile decks (first deck of 32.9 m^2^; second deck 29.10 m^2^) of the same transport company. Specifically, three different shipments, travelling on three different days one month apart from the other (February to April), transporting a consignment of about 30 animals each, were considered. More than half of the animals travelling in each shipment were included. Briefly, out of a total of 94 animals, in the first, second and third shipment (day1, day2, day3), 20/32, 15/29 and 21/33 animals were randomly tested, respectively. In France, before collection at the assembly point, animals were reared at pasture on different farms, located close to the assembly point. At the assembly point they were grouped respecting the original penning, were kept for less than 24 h, and then shipped to Italy. Before shipping, official veterinarians checked the health of the animals and those with clinical signs were not loaded. During the travel, steers received water and feed at regular intervals and no unloading stops were planned, in compliance with the EC Regulation 1/2005. Upon arrival, the animals included in the study were located separately (i.e., isolated in a different pen) to the other animals travelling together and the animals already reared at the arrival farm. During the study, the animals were fed with alfalfa barley silage mixed diets which were similar to the feed provided at the assembly point and during the journey.

Prior to shipment (T0), and four days after arrival (T1), two deep nasopharyngeal swabs (DNS)/animal, one swab in each nasal cavity, were collected from the 56 selected steer using sterile transport swabs of 13 cm (Nuova Aptaca SRL, Canelli, AT, Italy). Contextually, at T1, four days after the first shipment arrived, two DNS/each were collected from two animals located in the arrival farm to evaluate the epidemiological status of the residential herd. T0 DNS were kept at 4 °C during the journey and at the arrival farm. After T1 collection, all DNS were stored on ice, transported to the laboratory of Infectious Diseases of the Department of Veterinary Medicine of Bari (Italy), and processed within 24 h. No antimicrobials or vaccines were administered prior to or during the study to any of the steers tested.

Nucleic acids were extracted from each DNS using the commercial kit QIAamp^®^ Viral RNA Mini Kit (Qiagen S.p.A., Milan, Italy), according to the manufacturer’s instructions, and were stored at −80 °C until tested by Real Time quantitative PCR (qPCR). Primers and TaqMan probe for qPCR assay were used as previously described [13], in the same reaction conditions, including reaction mix component and thermal cycling (Supplementary Table S1). 

The tested pathogens were the following bacteria: *Pasteurella multocida*, *Mannheimia haemolytica*, *Histophilus somni*, and *Mycoplasma bovis*, and the following viral pathogens: BoHV-1, BAdV, BVDV, BCoV, BRSV and BPiV. Categorical dichotomous data regarding the outcome of qPCR for each pathogen detected in animals, considered as binary (positive/negative, 1/0), at different times (before and after journey, T0 and T1) and for different days (day1, day2 and day3) were described as counts and percentages. The relationship between the outcome of qPCR for each pathogen detected in animals as a dependent categorical dichotomous variable, defined as a dummy variable (positive/negative, 1/0), and the time (T0, T1) and day (day1, day2 and day3) as independent factors, were evaluated by univariate logistic regression model. The odds ratio (OR), confidence interval 95% (CI 95%) and *p*-values were assessed. *p* values were calculated using Wald Test. Statistical analysis was carried out using GenStat^®^ Version 14 (VSN International, Hemel Hempstead, UK).

## 3. Results

Table 1 shows the number of positive animals in each truck at T0 and T1 for each pathogen tested. Neither BVDV nor BoHV-1 were detected, either at T0 or T1.

There was an association between day and *H. somni*, and a higher prevalence was found in DNS collected from animals travelling in the third shipment (Day 3) (90%, 38/42) in comparison with the first (60%, 24/40) and second (60%, 18/30) days (OR = 6.3, 95% CI = 1.8–21.2, *p* = 0.006). There were no other changes in the prevalence of the other pathogens among days. 

The results of the univariate logistic regression analyses with time (before and after journey: T0 and T1, respectively) as a fixed factor are presented in Table 2. Higher odds of BCoV, BRSV. *M. haemolytica M. bovis*, and *P. multocida* were found in DNS collected four days after arrival (T1) compared with DNS collected before departure (T0). There was no difference in the prevalence of BAdV, BPiV, *H. somni* in the DNS collected at T0 and T1 (*p* > 0.05). 

The DNS collected from the two steers reared at the arriving farm in southern Italy during the period from February to April, resulted positive only for BCoV.

## 4. Discussion

This pilot study documented the prevalence of the multiple pathogens involved in BRD before and after a long-distance travel from France to southern Italy. Our results are in agreement with the current literature and, considering that the tested animals were not treated or vaccinated before and after the transport, the reported data support the hypothesis that the prevalence of pathogens related to BRD would increase after the tested journey. Interestingly, an increase in positivity towards BCoV, BRSV, *H. somni*, *P. multocida*, *M. haemolytica,* and *M. bovis* was observed, while neither BVDV nor BoHV-1 were ever detected. 

As already demonstrated [4], the nasal microbiota tested in the present study showed changes when the DNS before the transport and after the arrival at the feedlot in Italy were compared. Although the cattle involved in this study only originated from France feedlots, constraining our findings, the study has several strengths and points of interest. BCoV and, mostly, *H. somni*, were detected at T0 with fair frequency, indicating that, for these pathogens, some animals were already infected before loading. This datum requires clarifications. In the dissemination of BCoV infection, stressors such as the comingling or transport of cattle play a significant role in viral replication and shedding. However, though the shedding of the virus can be very long, it does not necessarily indicate transmission potential [14,15]. In a recent study, high BCoV arrival titers were detected in groups of animals on arrival at different feedlots, but titer changes after arrival reduced BRD risk, suggesting that the positivity on arrival could be expression of healthy animals, able to mount an effective immune response, rather than evidence of BCoV-specific protection per se [16]. On balance, epidemiological evidence of BCoV-induced BRD is persuasive, although the centrality of that role remains somewhat unclear.

In the third shipment (Day 3), the positivity for *H. somni* was higher than in the other two shipments considered. In recent studies, the most frequent bacterium isolated in the lower respiratory tract of cattle with BRD was *P. multocida*, followed by *M. haemolytica* and *H. somni*, and the identification of these three pathogens was more frequent in cattle with BRD compared to healthy cattle [17,18]. In addition, Welsh et al. [19] observed a variation in the ratio of *M. haemolytica* to *P. multocida* isolations from beef cattle with pneumonia in about six years. In the present study, *H. somni* was found to be the most common bacterium identified, whereas *P. multocida* and *M. haemolytica* were more rarely detected. The latter result may be related to a change in virulence among these pathogens due to antibiotic pressure and the development of bacterial resistance [18]. However, regardless of its origin, the high prevalence of *H. somni* required a more in-depth control of this pathogen in feedlot cattle, and more careful attention and investigation before shipping.

The other pathogens found were associated with time, with animals more likely to be found positive four days after the tested journey, thus, other factors, such as transport stress and the epidemiological status of the herd at arrival, may have played an important role. It is important to highlight that, in our study, DNS collected from the animals at the local farm were positive only for BCoV, thus the epidemiological status of the herd at arrival may be one of the reasons for the higher prevalence of this virus found in the DNS at T1. This datum supports the theory that the epidemiological status of arrival farms should be considered as a possible risk factor of BRD [4,20]. 

Overall, it is evident that the bovine nasopharyngeal microbiota undergoes changes following arrival at the feedlot and its evolution in beef cattle after arrival at a feedlot was also described by other researchers [4,20]. The feedlot environment, and its associated stressors, may provide conditions that allow for the proliferation of *M. bovis* in the nasopharynx within the first 14 days of feedlot placement [4]. Although feedlot placement did not increase BRD-associated pathogens, the relative variability of the microbiota observed following feedlot placement may explain why beef steers are most susceptible to BRD during this period. 

Our data should be considered preliminary because this study was limited by a number of factors. Firstly, due to the small number of events (three shipments, 112 samples) the logistic model was likely to suffer from small-sample bias. Secondly, the effects of other factors, such as age of the animals, driver, month, the number of farms per transport, climate conditions, space allowance and mingling were not considered, consequently, our results are valid only for the tested type of journey and animals. It is indeed well known that management conditions of the transport, distance travelled, season and geographic area of origin, are crucial elements in order to define the risk profile of developing BRD. Thirdly, the prevalence of the pathogens at the farms of origin and the number of farms per each shipment was not determined. This gap was due to the fact that the animals collected at the assembly point arrived from different local farms, and it was not possible to sample animals over there and we had no access to the documentation reporting the origin of each animal. However, sampling before loading provides a partial indication of the animal’s health status. Lastly, a great limitation of the study is that it was not possible to split the effects of the stress due to transport from stress due to the adaptation to a new farm and to document how those stressors affect the immune system of the animals. It has been found that potential causes for BRD onset include stress associated with transport, adaptation to feed, the new social hierarchy, and viral exposure [1,2]. It is well known that these stressors affect respiratory immunity, response to infection, bacterial growth, viral replication and the tissue repair process, and are responsible for an increase in cortisol release and a decrease in leukocyte numbers [20,21]. Consequently, it has been suggested that, to reduce the economic cost of the disease, it may be more effective to focus on the host than the pathogens [1,22]. Unfortunately, in this pilot study it was not possible to determine stress or other immunology biomarkers, thus further studies are needed to explain other possible causes of the increase in the prevalence of BRD pathogens in the nasal microbiome of beef steers after travelling. Our results need, therefore, to be confirmed in a larger dataset, including more events and evaluating the effects of multiple factors using multivariate regression analysis to provide useful guidelines to prevent the performance losses and poor welfare associated with BRD. Notwithstanding the aforementioned limitations, this preliminary study increased our knowledge of the prevalence of BRD in beef steers.

## 5. Conclusions

Overall, this study documented an increase in the prevalence of BRSV, BCoV, *P. multocida*, *M. haemolytica*, *H. somni*, *M. bovis* in the nasal microbiota of beef steers after transportation. However, this was a pilot study, testing the effects of the same journey from France to Italy, where some parameters, including the effect of driver, month, number of farms per transport, and climate conditions, were unclear. Consequently, further investigations on factors influencing microbiota composition during long distance travels, such as environmental parameters and journey management, should be performed to better define parameters that can alter the nasal microbiota in bovine.

## Figures and Tables

**Table 1:** Total number of animals found positive at departure (T0) and 4 days after arrival (T1) in each day of transport for the following pathogens: *bovine respiratory syncytial virus* (BRSV), *bovine adenovirus* (BAdV), *bovine coronavirus* (BCoV), *bovine parainfluenza virus* (BPiV), *Histophilus somni, Mannheimia haemolytica, Mycoplasma bovis, Pasteurella multocida.* Data are expressed as counts and percentages.

Pathogen	Time	Positive Outcomes (qPCR)		
		Day 1 n (%)	Day 2 n (%)	Day 3 n (%)
BRSV	T0	1/20 (5)	0/15 (0)	0/21 (0)
	T1	12/20 (60)	0/15 (0)	0/21 (0)
BAdV	T0	0/20 (0)	1/15 (6.6)	2/21 (9.5)
	T1	0/20 (0)	5/15 (33.3)	3/21 (14.3)
BCoV	T0	5/20 (25)	0/15 (0)	8/21 (38.1)
	T1	16/20 (80)	12/15 (80)	14/21 (66.6)
BPiV	T0	0/20 (0)	0/15 (0)	0/21 (0)
	T1	2/20 (10)	0/15 (0)	0/21 (0)
*H. somni*	T0	4/20 20)	3/15 (20)	17/21 (81)
	T1	20/20 (100)	15/15 (100)	21/21 (100)
*M. haemolytica*	T0	0/20 (0)	0/15 (0)	4/21 (19)
	T1	8/20 (40)	1/15 (6.6)	3/21 (14.3)
*M. bovis*	T0	0/20 (0)	3/15 (20)	5/21 (23.8)
	T1	0/20 (0)	13/15 (86.6)	7/21 (33.3)
*P. multocida*	T0	3/20 (15)	3/15 (20)	5/21 (23.8)
	T1	12/20 (60)	13/15 (86.6)	7/21 (33.3)

**Table 2:** Results of univariate logistic regression analyses of associations between presence of pathogens related to bovine respiratory disease (BRD) in the deep nasopharyngeal swabs (DNS) collected before (T0) and four days after a long journey (T1) in the 56 steer beef analyzed (OR: odds ratios; CI: confidence intervals; *p*: Wald test *p* value).

Pathogen	Time	Positive % (n)	OR	95%CI	*p*
BRSV	T0	1.8 (1/56)	Ref		
	T1	21.4 (12/56)	15.0	1.9–117.6	0.010
BCoV	T0	23.2 (13/56)	Ref		
	T1	75.0 (42/56)	9.9	4.1–23.5	<0.001
*M. haemolytica*	T0	7.1 (4/56)	Ref		
	T1	21.4 (12/56)	3.5	1.1–11.7	0.039
*M. bovis*	T0	12.5 (7/56)	Ref		
	T1	35.7 (20/56)	3.8	1.5–10.2	0.006
*P. multocida*	T0	19.6 (11/56)	Ref		
	T1	57.1 (32/56)	5.4	2.3–12.7	<0.001

## Supplementary Materials

The following are available online at https://www.mdpi.com/2076-2615/9/12/1093/s1, Table S1, Primer and probe sets for the detection of BRD pathogens.
