# Supplementary material for: Prevalence of Pathogens Related to Bovine Respiratory Disease Before and After Transportation in Beef Steers: Preliminary Results
Source: Animals (Basel). 2019 Dec 6;9(12):1093. doi: 10.3390/ani9121093 (PMC6940923; doi:10.3390/ani9121093)
Supplement: Supplementary file 1 [file animals-09-01093-s001.pdf]

Table S1. Primer and probe sets for the detection of BRD pathogens

Pathogens	5'>3' F	5'>3'R	5'>3' FAM
**BPIV**	TGTCTTCCACTAGATAGA	GCAATGATAACAGGACTA	ACAGCAATTGGATCAATA0[MGBEQ]
**BVDV**	GGGNAGTCGTCARTGGT	GTGCCCATGTACAGCAGAGWTTTT	CCAYGTGGACGAGGGCAYGC[TAM]
**BCoV**	GGACCCAAGTAGCGATGAG	GACCTTCCTGAGCCTTCAATA	ATTCCGACTAGGTTTCCGCCTGG[TAM]
**BRSV**	GCAATGCTGCAGGACTAGGTATAAT	ACACTGTAATTGATGACCCCATTCT	ACCAAGACTTGTATGATGCTGCCAAAGCA[TAM]
**BoHV1**	CAATAACAGCGTAGACCTGGTC	GCTGTAGTCCCAAGCTTCCAC	TGCGGCCTCCGGGCTTTACGTCT[TAM]
**BAdV3**	ATTACCAGCGTCAACCTCTAC	CCGCCGAGAGATAGTCATTAAA	TCCACTTTGGAAGCTATGCTCCGC[TAM]
M. haemolytica	ATTAGTGGGTTGTCCTGGTTAG	GCGTGATTTCGGTTCAGTTG	CTGAACCAACACGAGTAGTCGCTGC[TAM]
P. multocida	GGGCTTGTCGGTAGTCTTT	CGGCAAATAACAATAAGCTGAGTA	CGGCGCAACTGATTGGACGTTATT[TAM]
H. somni	AAGGCCTTCGGGTTGTAAAG	CCGGTGCTTCTTCTGTGATTAT	CGGTGATGAGGAAGGCGATTAG[TAM]
M. bovis	TCAAGGAACCCCACCAGAT	AGGCAAAGTCATTTCTAGGTGCAA	TGGCAAACTTACCTATCGGTGACCCT[TAM]
